# Redifferentiation of Adult Human β Cells Expanded *In Vitro* by Inhibition of the WNT Pathway

**DOI:** 10.1371/journal.pone.0112914

**Published:** 2014-11-13

**Authors:** Ayelet Lenz, Ginat Toren-Haritan, Shimon Efrat

**Affiliations:** Department of Human Molecular Genetics and Biochemistry, Sackler School of Medicine, Tel Aviv University, Tel Aviv, Israel; University of Alabama at Birmingham, United States of America

## Abstract

In vitro expansion of adult human islet β cells is an attractive solution for the shortage of tissue for cell replacement therapy of type 1 diabetes. Using a lineage tracing approach we have demonstrated that β-cell-derived (BCD) cells rapidly dedifferentiate in culture and can proliferate for up to 16 population doublings. Dedifferentiation is associated with changes resembling epithelial-mesenchymal transition (EMT). The WNT pathway has been shown to induce EMT and plays key roles in regulating replication and differentiation in many cell types. Here we show that BCD cell dedifferentiation is associated with β-catenin translocation into the nucleus and activation of the WNT pathway. Inhibition of β-catenin expression in expanded BCD cells using short hairpin RNA resulted in growth arrest, mesenchymal-epithelial transition, and redifferentiation, as judged by activation of β-cell gene expression. Furthermore, inhibition of β-catenin expression synergized with redifferentiation induced by a combination of soluble factors, as judged by an increase in the number of C-peptide-positive cells. Simultaneous inhibition of the WNT and NOTCH pathways also resulted in a synergistic effect on redifferentiation. These findings, which were reproducible in cells derived from multiple human donors, suggest that inhibition of the WNT pathway may contribute to a therapeutically applicable way for generation of functional insulin-producing cells following ex-vivo expansion.

## Introduction

Beta-cell replacement represents a promising approach of treatment of type 1 diabetes. However, tissue donor shortage remains a major obstacle. In-vitro expansion of functional β-cells from adult human islets is an attractive approach for generating an abundant source of cells for β-cell replacement, however cell proliferation is associated with a rapid loss of β-cell phenotype [1]–[3]. Using a genetic lineage-tracing approach based on lentivirus vectors we provided evidence for massive proliferation and dedifferentiation of human β-cell-derived (BCD) cells [4], which is associated with a process similar to epithelial-mesenchymal transition (EMT) [5]. BCD cells, which constitute ∼40% of islet cell cultures [4], maintain open chromatin structure at β-cell genes [6] and can be redifferentiated in response to a combination of soluble factors termed Redifferentiation Cocktail (RC) [7]. However, RC treatment leads to redifferentiation of only a fraction of BCD cells. In search for improved redifferentiation approaches, we analyzed changes in signaling pathways involved in EMT during dedifferentiation of BCD cells. We have shown that the NOTCH pathway is activated during this process [8], and its inhibition by shRNA against the NOTCH pathway mediator HES1 results in enhanced BCD cell redifferentiation [9]. Here we investigated the role of the WNT pathway in BCD cell dedifferentiation and redifferentiation.

In the canonical WNT pathway, WNT ligands regulate gene transcription by controlling protein levels and nuclear localization of β-catenin. In the absence of WNT signaling, glycogen synthase kinase 3β (GSK3β), adenomatous polyposis coli protein (APC), AXIN, and β-catenin form a destruction complex, enabling GSK3β to phosphorylate β-catenin and target it to degradation [10]. Under these conditions most of cellular β-catenin is localized to the plasma membrane in complex with E-cadherin (CDH1), a calcium-dependent intercellular adhesion protein required for epithelial cell polarity and identity [11]. This complex is required for association of epithelial adherens junctions with the cytoskeleton [12]. WNT ligand binding to the frizzled (FZD)/LRP5/6 receptor complex blocks destruction complex activity by inhibiting GSK3β [13]. This enables accumulation and nuclear translocation of β-catenin. Once inside the nucleus, β-catenin acts in combination with other transcription factors to stimulate transcription of WNT-responsive genes [13]. WNT pathway signaling downregulates CDH1 by activating expression of SNAI1, SNAI2 and TWIST, which block *CDH1* transcription [12]. Loss of ECAD, and activation of N-cadherin (CDH2), are hallmarks of EMT.

WNT pathway activity is necessary for normal pancreas development [14]–[17], however, in adult β-cells the pathway is normally downregulated [16], [17]. Several studies have shown that WNT pathway activation induces mouse islet cell proliferation [18], [19]. In addition, *Ccnd1* and *Myc*, both of which are WNT target genes [20], [21], were shown to induce β-cell proliferation [22], [23]. Furthermore, human islet cell proliferation in-vitro was induced by activation of the WNT/β-catenin signaling pathway through inhibition of GSK3β [24].

In this work we analyzed the role of WNT pathway signaling in BCD cell proliferation and dedifferentiation. Our findings demonstrate that the WNT pathway is activated in BCD cells, while inhibition of β-catenin expression by shRNA leads to BCD cell growth arrest and redifferentiation. β-catenin inhibition synergizes with RC and *HES1* shRNA treatments, resulting in enhanced cell redifferentiation.

## Materials and Methods

### Ethics statement

This study was conducted according to the principles expressed in the Declaration of Helsinki. The Institutional Review Boards of the following medical centers, which provided human islets, each provided approval for the collection of samples and subsequent analysis: University of Geneva School of Medicine; San Raffaele Hospital, Milan; Faculty of Medicine, Lille 2 University; Massachusetts General Hospital; Washington University; University of Pennsylvania; Scharp/Lacy Institute; University of Illinois; University of Wisconsin; University of Miami; Southern California Islet Consortium. All donors provided written informed consent for the collection of all samples and subsequent analysis.

### Cell culture

Human Islets were received 2–6 days following isolation. Islets from individual donors (Table 1) were dissociated into single cells and cultured in CMRL 1066 medium containing 5.6 mmol/liter glucose and supplemented with 10% fetal bovine serum (FBS), 100 units/ml penicillin, 100 µg/ml streptomycin, 100 µg/ml gentamycin, and 5 µg/ml amphotericin B as described [3]. The cultures were refed twice a week and split 1∶2 once a week. Lineage tracing was performed using the RIP-Cre/ER and pTrip–loxP-NEO-STOP-loxP-eGFP lentiviral vectors as described [5]. 4-hydroxytamoxifen (Sigma-Aldrich) was added to a final concentration of 1 µM one day post-infection. Following overnight incubation the medium was changed to regular growth medium. Labeled cells were sorted using a FACS Aria sorter (BD Biosciences) as described [5].

**Table 1:** Donors of islets used in the study.

Donor no.	Donor sex	Donor age (y)	Donor BMI	Islet purity (%)
1	m	42	23.4	80
2	m	29	26.3	70
3	m	40	29.0	95
4	f	47	33.2	70
5	m	70	26.0	80
6	m	41	32.6	70
7	f	33	31.8	70
8	m	27	19.0	85
9	f	63	23.4	70
10	m	62	26.6	81
11	m	48	18.0	90
12	m	45	26.7	85
13	m	54	33.0	85
14	f	54	29.4	83
15	f	66	23.5	82
16	m	32	25.7	70
17	m	52	21.2	80
18	f	39	21.9	90
19	f	51	21.2	85
20	f	41	35.5	90
21	m	27	20.2	85
22	f	37	23.8	80
23	f	32	26.9	80
24	f	50	42.3	85
25	m	21	33.8	85
26	m	38	29.8	85
27	m	29	30.2	95
28	f	49	27.1	90
29	f	58	26.7	99
30	f	46	33.0	80
31	m	34	27.8	85
32	m	46	24.0	74
33	f	44	34.5	95
34	f	47	20.6	90
35	m	62	18.8	92
36	m	31	29.0	85
37	m	55	22.4	70
38	f	38	24.0	75
39	m	56	30.8	90
40	m	54	26.2	80
41	f	64	30.0	85
42	f	51	39.3	80
43	m	27	19.0	85
44	m	49	31.1	90
45	f	48	22.0	95
46	m	14	27.1	80
47	F	48	32.2	85
48	F	47	22.5	70
49	m	59	24.5	85
50	m	54	29.1	93
Mean±SD		45±12	27±5	83±8

### β-catenin inhibition by shRNA

β-catenin shRNAs (accession numbers TRCN-3843, -3844,-3845,-3846 and -10824) and a non-target shRNA cloned in plko.1-PURO lentiviral vector were obtained from the RNAi Consortium (Sigma-Aldrich). Virus was produced in 293T cells as described [5]. Cells were infected at MOI 2∶1 in CMRL 1066 medium containing 8 µg/ml polybrene overnight. The medium was then replaced with culture medium. Four days following infection the cells were selected with 1 µg/ml puromycin for 3 days. All shRNAs were evaluated for β-catenin inhibition in HeLa cells before use in expanded islet cells.

### Redifferentiation of expanded islet cells

Expanded human islet cells or sorted BCD cells in passages 5–7 were infected with β-catenin shRNA or nontarget shRNA viruses. Five to 6 days following infection cells were trypsinized, pelleted, and seeded at 3.8×104 cells/cm2 in ultra-low attachment plates (Corning) in CMRL 1066 medium containing 5.6 mM glucose and supplemented with 100 U/ml penicillin, 100 µg/ml streptomycin, 100 µg/ml gentamycin sulphate (Biological Industries), 1% BSA fraction V (Sigma), 1× insulin/transferrin/selenium (ITS, Invitrogen), D-Glucose (final concentration 25 mM), 8 nM exendin-4 (Acris), 8 nM activin A (Cytolab/PreproTech), 1× B27 supplement (Stem Cell Technologies), and 10 mM nicotinamide (Sigma) (Redifferentiation Cocktail, RC) for the indicated periods. Half-volume medium changes were performed every other day.

### qPCR analysis

Total RNA was extracted using the High Pure RNA isolation kit (Roche), or with ZR RNA MiniPrep RNA Isolation Kit (Zymo), and treated with RNase-free DNase1 (Thermo), or with TRI©Reagent (Sigma) followed by treatment with DNA-Free (Ambion). cDNA was produced using SuperScript III First-Strand Synthesis System (Invitrogen) or High-Capacity cDNA Reverse Transcription Kit (Applied Biosystems). qPCR was carried out in triplicates using the Universal Probe Library System (Roche) in 7300 Real-time PCR system (Applied Biosystems). Results were normalized to the TATA-box-binding protein (TBP) or Ribosomal protein large P0 (RPLP0) transcripts. These genes were selected as normalization controls since their detection threshold occurred at the same cycle in all the samples studied. Data analysis was performed with qBase software. Table 2 lists primer sequences. All reactions were performed with annealing at 60°C for 40 cycles. For undetectable transcripts, the cycle number was set to 40 for comparisons.

**Table 2:** Primer sequences for qPCR analyses.

Gene symbol	Sense primer	Antisense primer
*ABCC8*	AGACCCTCATGAACCGACAG	GGCTCTGTGGCTTTTCTCTC
*ACTA2*	GCTTTCAGCTTCCCTGAACA	GGAGCTGCTTCACAGGATTC
*AKT1*	GGCTGAAGAGATGGAGGTGT	GGATCACCTTGCCGAAAGT
*AXIN2*	CCACACCCTTCTCCAATCC	TGCCAGTTTCTTTGGCTCTT
*CCND1*	GAAGATCGTCGCCACCTG	GACCTCCTCCTCGCACTTCT
*CDKN1A*	CCGAAGTCAGTTCCTTGTGG	CATGGGTTCTGACGGACAT
*CDKN1B*	TTTGACTTGCATGAAGAGAAGC	AGCTGTCTCTGAAAGGGACATT
*CDKN1C*	CTCCTTTCCCCTTCTTCTCG	TCCATCGTGGATGTGCTG
*CDKN2A*	GTGGACCTGGCTGAGGAG	TCTTTCAATCGGGGATGTCT
*CTNNB1*	GCTTTCAGTTGAGCTGACCA	CAAGTCCAAGATCAGCAGTCTC
*CDH1*	GCCGAGAGCTACACGTTCA	GACCGGTGCAATCTTCAAA
*CDH2*	CTCCATGTGCCGGATAGC	CGATTTCACCAGAAGCCTCTAC
*FZD2*	GGTGTCGGTGGCCTACAT	GAGAAGCGCTCGTTGCAC
*GCG*	GTACAAGGCAGCTGGCAAC	TGGGAAGCTGAGAATGATCTG
*GCK*	GCAGATGCTGGACGACAG	TCCTGCAGCTGGAACTCTG
*HAPLN1*	CCTGGATTTCAGGACAAGTGA	TCCAGAGTATAGTTGTCTGAAAGATG
*HES1*	TTACGGCGGACTCCATGT	AGAGGTGGGTTGGGGAGT
*IAPP*	TTACCAAATTGTAGAGGCTTTCG	CCCTGCCTCTATACACTCACTACC
*ICAM1*	AGTGATCAGGGTCCTGCAA	GGGAGGGAGTCCTCCAATAC
*INS*	AGGCTTCTTCTACACACCCAAG	CACAATGCCACGCTTCTG
*ITGB8*	TTTGCAGCATCTTACATGTCTTG	TGTTTTTCACAGCACTGATTGTT
*JAG1*	GCGTGCTGGGTAGAGGTG	GAGAAGGACCCGGAGAGC
*JAG2*	AAACCTGATTGGCGGCTATT	TGACGTTGATATGGCAGTTGA
*KCNJ11*	TGTGTCACCAGCATCCACTC	CACTTGGACCTCAATGGAGAA
*LMOD1*	GGAAGATGGGAGACAAAGTCC	ACTGAAGCAGTTTGGGCACT
*MAFA*	AGCGAGAAGTGCCAACTCC	TTGTACAGGTCCCGCTCTTT
*MNX1*	TGCCTAAGATGCCCGACTT	AGCTGCTGGCTGGTGAAG
*MYC*	CACCAGCAGCGACTCTGA	GATCCAGACTCTGACCTTTTGC
*NEUROD1*	CTGCTCAGGACCTACTAACAACAA	GTCCAGCTTGGAGGACCTT
*NKX2.2*	CGAGGGCCTTCAGTACTCC	GGGGACTTGGAGCTTGAGT
*NKX6.1*	CGTTGGGGATGACAGAGAGT	CGAGTCCTGCTTCTTCTTGG
*NOTCH1*	CGCACAAGGTGTCTTCCAG	AGGATCAGTGGCGTCGTG
*NOTCH2*	GGCAGACTGGTGACTTCACTT	CTCTCACAGGTGCTCCCTTC
*NOTCH4*	TCTCCCTGTGCCAATGGT	AGGCACTCATCCACCTCTGT
*PCSK1*	TGATCCCACAAACGAGAACA	TGTGATTATTTGCTTGCATGG
*PDX1*	CACATCCCTGCCCTCCTAC	GAAGAGCCGGCTTCTCTAAAC
*PITX2*	CCTTACGGAAGCCCGAGT	CCGAAGCCATTCTTGCATA
*PPY*	TCTAGTGCCCATTTACTCTGGAC	GCAGGTGGACAGGAGCAG
*RPLPO*	TCTACAACCCTGAAGTGCTTGAT	CAATCTGCAGACAGACACTGG
*SCG5*	CATACCGCAGTAGGCTCCTC	GCCAGATAGCATGGTAGAGACC
*SIRT1*	AAATGCTGGCCTAATAGAGTGG	TGGCAAAAACAGATACTGATTACC
*SNAI1*	TGGTTGCTTCAAGGACACAT	GTTGCAGTGAGGGCAAGAA
*SNAI2*	GCTGCAGGACTCTAATCCAGA	ATCTCCGGAGGTGGGATG
*SOD2*	GCACTAGCAGCATGTTGAGC	CCGTAGTCGTAGGGCAGGT
*SST*	ACCCCAGACTCCGTCAGTTT	ACAGCAGCTCTGCCAAGAAG
*SYT11*	ACCAATATCCGACCTAGCTTTG	GACACACACCACCAGCACA
*TBP*	CGGCTGTTTAACTTCGCTTC	CACACGCCAAGAAACAGTGA
*THY1*	AGGAGCCGGACACTTCTCA	AGTCACAGAACAGGAAGAACCAC
*TWIST1*	AAGGCATCACTATGGACTTTCTCT	GCCAGTTTGATCCCAGTATTTT

### Immunofluorescence analysis

Cells were spotted on slides using Shandon Cytospin4 centrifuge (Thermo Scientific), and fixed for 15 minutes at room temperature in 4% paraformaldehyde. For nuclear antigens, cells were incubated in 0.25% NP40 for 10 minutes at room temperature prior to blocking. Samples were blocked for 15 min at room temperature in blocking buffer (1% BSA, 10% fetal goat serum, and 0.2% saponin) and incubated overnight at 4°C with primary antibodies diluted in blocking buffer as follows: mouse anti-β-catenin (1∶200, Cell Signaling); mouse anti-active β-catenin (1∶500, Millipore); rat anti-human C-peptide (1∶1000, Beta Cell Biology Consortium); rabbit anti-eGFP (1∶1000, Invitrogen); rabbit anti-Ki67 (1∶200, Zymed); mouse anti-Ki67 (1∶200, Zymed); and mouse anti- human PDX1 (1∶500, R & D Systems). Slides were washed in blocking buffer 5 times, and incubated with a secondary antibody conjugated to Alexa fluorophores (1∶1000, all from Invitrogen,). DNA was stained with DAPI. The slides were mounted with Fluorescent Mounting Medium (GBI Labs). Images were visualized under a fluorescent BX61 microscope or TCS SP5 confocal fluorescent microscope. To demonstrate antibody specificity, a minus-primary antibody control was employed.

### Immunoblotting

Cellular protein was extracted for 10 min in 50 mM Tris-HCl buffer, pH 7.4, containing 0.5% NP-40, 0.7% NaCl, 0.2% EDTA, and protease inhibitors. Samples of 20–40 µg protein were resolved by SDS-PAGE and transferred to Immobilon-P Membrane (Millipore). Non-specific sites were blocked for one hour at room temperature in blocking buffer containing 5% skim milk in TTBS buffer. The membrane was then incubated with rabbit anti-β-catenin (1∶10000, Abcam), mouse anti-SNAI2 (1∶200, Abgent), rabbit anti-FOXO1 (1∶1000, Cell Signaling), or rabbit anti-phospho FOXO1 (1∶1000, Cell Signaling). The bound antibody was visualized with appropriate horseradish peroxidase-conjugated anti-IgG (Jackson) and SuperSignal West Chemiluminescent Substrate kit (Pierce). Signal intensity was quantitated using TINA software.

### Apoptosis detection assay

TUNEL assay was performed using In Situ Cell Death Detection Kit (Roche), according to the manufacturer's protocol. The fluorescence was visualized under a fluorescent BX61 microscope.

### DNA microarray analysis

Total RNA was isolated as above. Hybridization to Affymetrix GeneChip Human Gene 1.0 ST Arrays, washing, and scanning were performed according to the manufacturer. Microarray analysis was performed on CEL files using Partek Genomics Suite TM (Partek). Data were normalized with the multi-average method. Batch effect removal was applied for the different samples, followed by one-way ANOVA. Clustering analysis was performed by Partek Genomics Suite software with Pearson's dissimilarity correlation by average linkage methods. The raw data has been deposited in the GEO database (accession number GSE60803).

### Statistical Analysis

Significance was determined using Student's t test. To approach a normal distribution of the qPCR data, a logarithmic transformation was performed. To account for multiple testing, the Bonferroni correction was applied.

## Results

**Figure 1:**
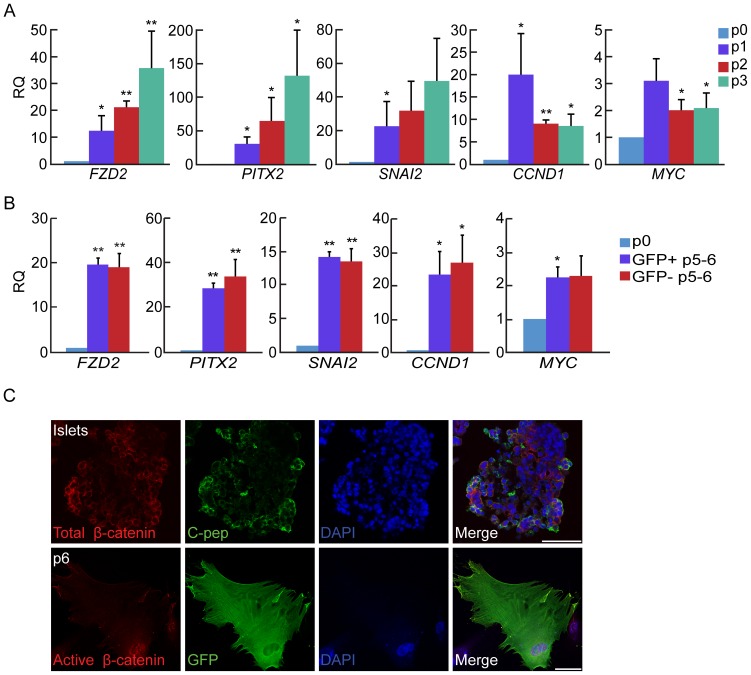
Upregulation of the WNT pathway in BCD cells. **A**, qPCR analysis of RNA extracted from isolated islets (p0) and expanded islet cells at the indicated passage number. **B**, qPCR analysis of RNA extracted from sorted eGFP^+^ BCD cells at passages 5–6. Data in A and C are mean±SE (n = 3–5 donors). *p<0.05, **p<0.005, compared with P0. **C**, Immunofluorescence analysis of isolated islets, and expanded islet cells at passage 6. Beta-catenin is localized in the membrane region in >98% of islet cells, while >98% of cells at passage 6 show β-catenin nuclear localization. Active β-catenin was not detected in islet β cells. Bar = 50 µm.

Analysis of WNT pathway gene expression during the first three weeks of islet cell expansion in vitro revealed a significant upregulation of transcripts encoding WNT pathway receptor and target genes (Fig. 1A). Transcripts for the WNT receptor FZD2 were upregulated >30-fold. Activation of the WNT pathway resulted in upregulation of its target genes *CCND1* and *MYC* (20-fold and 3-fold, respectively), which stimulate cell proliferation; *PITX2* (130-fold), which activates *CCND1* and *MYC*; and *SNAI2* (49-fold), which participates in inhibition of *CDH1* expression. Analysis of sorted eGFP^+^ cells, labeled by an insulin promoter-driven lineage tracing system [4], confirmed the activation of these genes in BCD cells (Fig. 1B). A comparable activation was observed in eGFP-negative cells, which include both non-BCD cells and BCD cells that were not labeled by eGFP [4]. Immunofluorescence analysis showed translocation of β-catenin from its membrane position in islet β-cells into the nucleus in eGFP^+^-labeled BCD cells (Fig. 1C), where it can activate transcription of target genes. The translocation was associated with a notable epithelial-to-mesenchymal morphological change.

**Figure 2:**
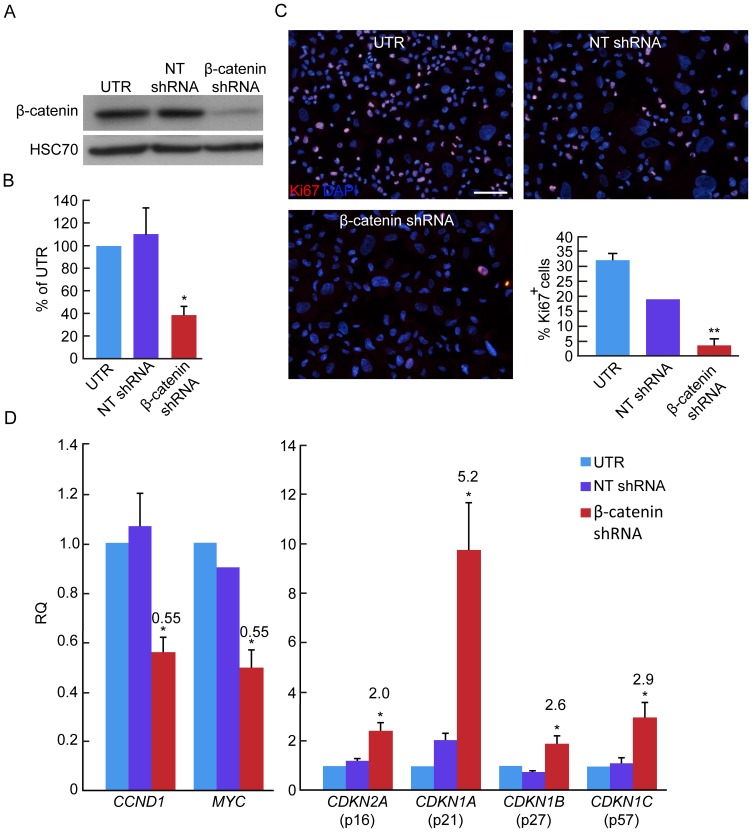
Effect of β-catenin downregulation on islet cell proliferation. **A**,**B**, Immunoblotting of β-catenin in expanded islet cells infected at passage 5 with β-catenin or nontarget (NT) shRNA viruses. HSC70 was used as a loading control. Data are mean±SE (n = 3 donors), shown as percentage of untreated (UTR) cells. *p<0.005, compared with NT shRNA. **C**. Immunofluorescence of Ki67 in expanded islet cells infected at passages 5–6 with β-catenin or NT shRNA viruses, and analyzed 7 days later. Values are mean±SD (n = 3 donors), based on counting >1000 cells per donor. **p<0.005, relative NT shRNA. Bar = 50 µm. **D**, qPCR analysis of RNA extracted from expanded islet cells at passages 5–6, 7 days following infection with β-catenin or NT shRNA viruses. Data are mean±SE (n = 3–4 donors). * p<0.05, relative to NT shRNA. Values on top of bars indicate fold change relative to NT shRNA.

To investigate the hypothesis that downregulation of WNT pathway in expanded BCD cells may result in their redifferentiation, we employed β-catenin shRNA to block its expression and prevent activation of WNT pathway target genes. Out of five shRNAs evaluated for their inhibitory activity, shRNA TRCN-3845 was selected for further analysis. This shRNA reduced β-catenin protein levels in expanded islet cells by >60%, compared with cells treated with nontarget (NT) shRNA (Fig. 2A,B). Analysis of cells infected with β-catenin shRNA virus 7-days post-infection revealed a great reduction in cell proliferation rate, as judged by Ki67 staining, to 12% of the rate in untreated cells (Fig. 2C). Transcript levels of β-catenin target genes *CCND1* and *MYC* decreased 2-fold, and transcripts encoding the cell cycle inhibitors p16, p21, p27, and p57, were upregulated 2–5-fold (Fig. 2D). Thus, inhibition of β-catenin expression resulted in a marked reduction in replication of cultured islet cells. This reduced replication rate did not correlate with an increase in cell apoptosis, as judged by terminal uridine deoxynucleotidyl transferase dUTP nick end labeling (TUNEL) assay (Fig. S1).

**Figure 3:**
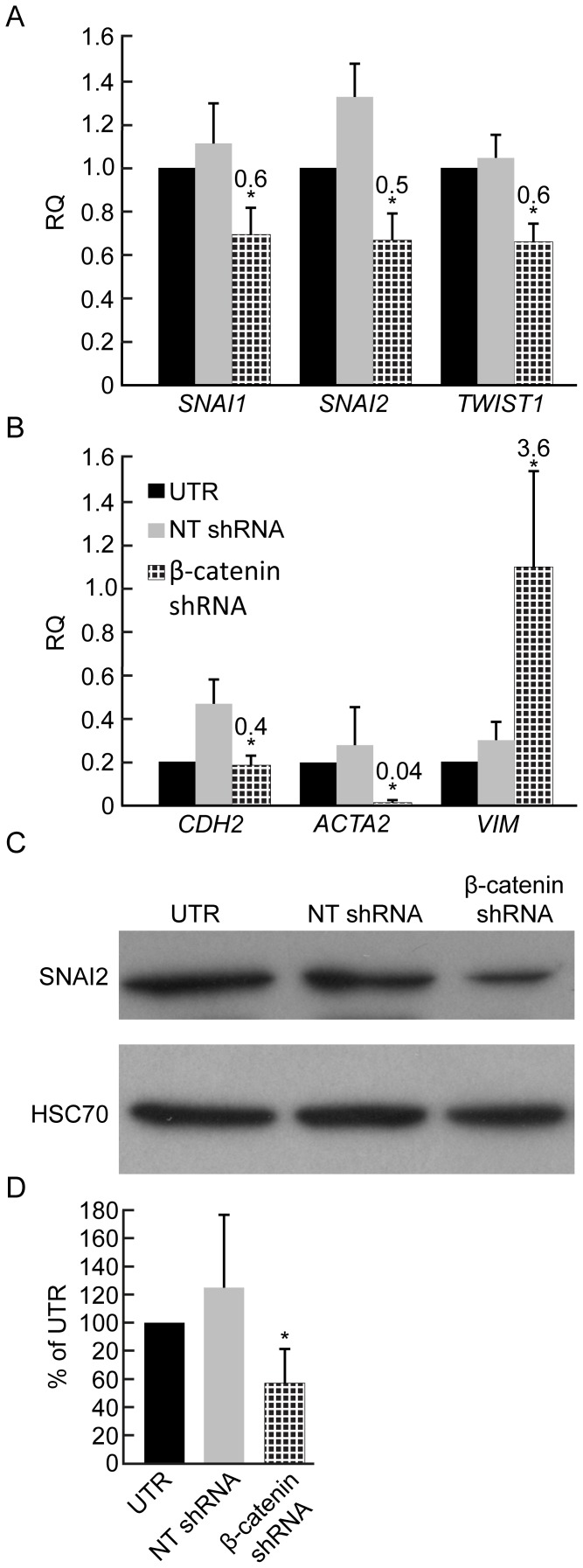
Effect of β-catenin downregulation on EMT marker gene expression in expanded islet cells. **A**,**B**, qPCR analysis of RNA extracted from expanded islet cells at passages 5–6, 7 days following infection with β-catenin or NT shRNA viruses. Data are mean±SE (n = 3–4 donors). * p<0.05, ** p<0.005, relative to NT shRNA. **C**,**D**, Immunoblotting of SNAI2 in expanded islet cells infected at passage 5 with β-catenin or NT shRNA viruses, and analyzed 7 days later. Data are mean±SE (n = 3 donors). *p<0.05.

We next analyzed the effects of β-catenin inhibition on the mesenchymal phenotype of expanded islet cells. *SNAI*2 transcript levels (Fig. 1A), as well SNAI2 protein levels (Fig. 3C,D), were reduced by 50%. In addition, transcripts encoding two other negative regulators of *CDH1*, SNAI1 and TWIST1, were also downregulated (Fig. 3A). A significant decrease was detected in transcripts for the mesenchymal markers NCAD and αSMA (Fig. 3B). In contrast, *VIM* transcripts were upregulated (Fig. 3B). Based on the majority of markers, we conclude that inhibition of β-catenin expression in expanded islet cells resulted in mesenchymal-epithelial transition (MET).

**Figure 4:**
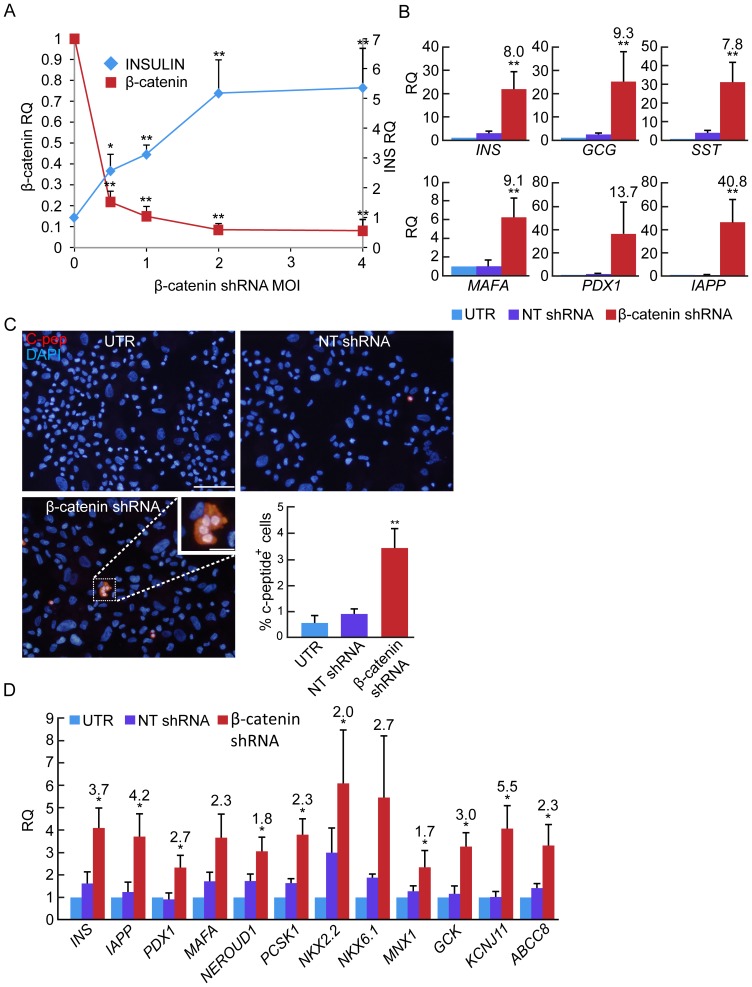
Effect of β-catenin downregulation on BCD cell redifferentiation. **A**, qPCR analysis of RNA extracted from expanded islet cells at passages 4–5, 7 days following infection with increasing amounts of β-catenin or NT shRNA viruses. Data are mean±SE (n = 3 donors). *p<0.05, **p<0.005, relative to NT shRNA. **B**, qPCR analysis of RNA extracted from expanded islet cells at passages 5–7, 7 days following infection with β-catenin or NT shRNA viruses. Data are mean±SE (n = 3–8 donors). *p<0.05, **p<0.005, relative NT shRNA. **C**, C-peptide immunofluorescence in expanded islet cells infected at passages 5–7 with β-catenin or NT shRNA viruses, and analyzed 7 days later. Bar = 50 µm Values are mean±SD (n = 4 donors), based on quantitation of >1000 cells in each group. *p<0.005, relative NT shRNA. **D**, qPCR analysis of RNA extracted from sorted eGFP^+^ BCD cells at passages 6–8, 7 days following infection with β-catenin or NT shRNA viruses. Data are mean±SE (n = 3–4 donors). *p<0.05, relative to NT shRNA.

**Figure 5:**
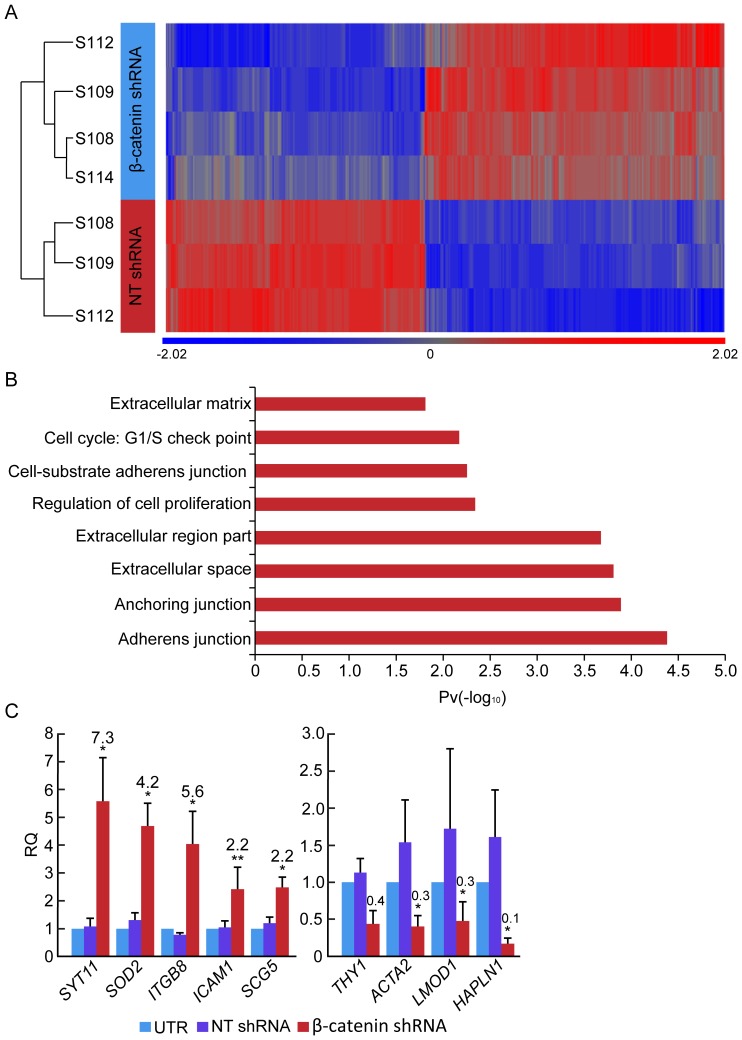
Global changes in gene expression in BCD cells infected with β-catenin shRNA. **A**, Heat map of cDNA microarray analysis of RNA extracted from sorted eGFP^+^ BCD cells at passages 4–5, 7 days following infection with β-catenin or NT shRNA viruses. n = 4 donors for β-catenin shRNA; n = 3 donors for NT shRNA. **B**, Gene ontology analysis of cDNA microarray results. **C**, Validation of candidate genes from the microarray analysis by qPCR analysis of RNA extracted from eGFP^+^ BCD cells at passages 5–6, 7 days following infection with β-catenin or NT shRNA viruses. Data are mean±SE (n = 3–4 donors). *p<0.05, **p<0.005, relative to NT shRNA. Validation of IAPP transcripts is seen in D.

Analysis of β-cell gene expression revealed a 8-fold increase in insulin transcript levels (Fig. 4A,B). The levels of insulin transcripts were inversely proportional to the levels of β-catenin transcripts, which were a function of the β-catenin shRNA virus MOI (Fig. 4A). This increase was manifested in a >3.5-fold elevation in the number of C-peptide^+^ cells, compared with cells treated with NT shRNA (Fig. 4C). This change reflected induction of redifferentiation in cells which did not express detectable insulin levels in the presence of high β-catenin levels, rather than upregulation of insulin expression in the minority of cells (∼0.5%) which did not undergo dedifferentiation. The increase in insulin transcripts was accompanied by induction of transcripts encoding the insulin gene transcription factors PDX1 and MAFA, as well as *IAPP* transcripts (Fig. 4B). In addition, *GCG* and *SST* transcripts were also elevated in expanded islet cells infected with the β-catenin shRNA virus (Fig. 4B). Our previous results have documented the potential of expanded islet cells to give rise to GCG- and SST-positive cells, which are distinct from insulin-positive cells generated in these cultures, and likely originate from non-BCD cells [7]. Analysis of RNA extracted from sorted eGFP^+^ BCD cells confirmed the activation of β-cell transcripts in BCD cells (Fig. 4D), and the lack of activation of *GCG*, *SST*, or *PPY* transcripts (Fig. S2). To profile global changes in gene expression induced in BCD cells by reducing β-catenin expression, RNA extracted from sorted eGFP^+^ cells infected with β-catenin or NT shRNA viruses was subjected to cDNA microarray analysis. A total of 297 genes were upregulated >1.5-fold, while 257 genes were downregulated >1.5-fold (Fig. 5A). Gene ontology analyses identified expression of genes involved in cell proliferation, cell adhesion, and cell-extracellular matrix interactions to have changed most significatly (Fig. 5B). The microarray results were validated by qPCR analyses of selected genes (Table 3, Fig. 5C). Taken together, these findings suggest that downregulation of β-catenin levels alone induces profound phenotypic changes in BCD cells, including growth arrest, MET, and redifferentiation.

**Table 3:** Genes selected for validation of microarray results by qPCR.

Gene symbol	Function	FC
*SYT11*	Regulated secretion	2.8
*SOD2*	Free-radical scavenger; high in islets vs. BCD cells	2.8
*ITGB8*	Cell-cell/extracellular matrix interaction	2.7
*ICAM1*	Cell adhesion	1.9
*SCG5*	Molecular chaperone of PC2; high in islets vs. BCD cells	1.8
*THY1*	Mesenchymal marker (CD90)	−1.7
*ACTA2*	Mesenchymal marker (αSMA)	−1.9
*LMOD1*	Actin binding	−2.0
*HAPLN1*	Cell adhesion; stability of extracellular matrix; low in islets vs. BCD cells	−3.3

**Figure 6:**
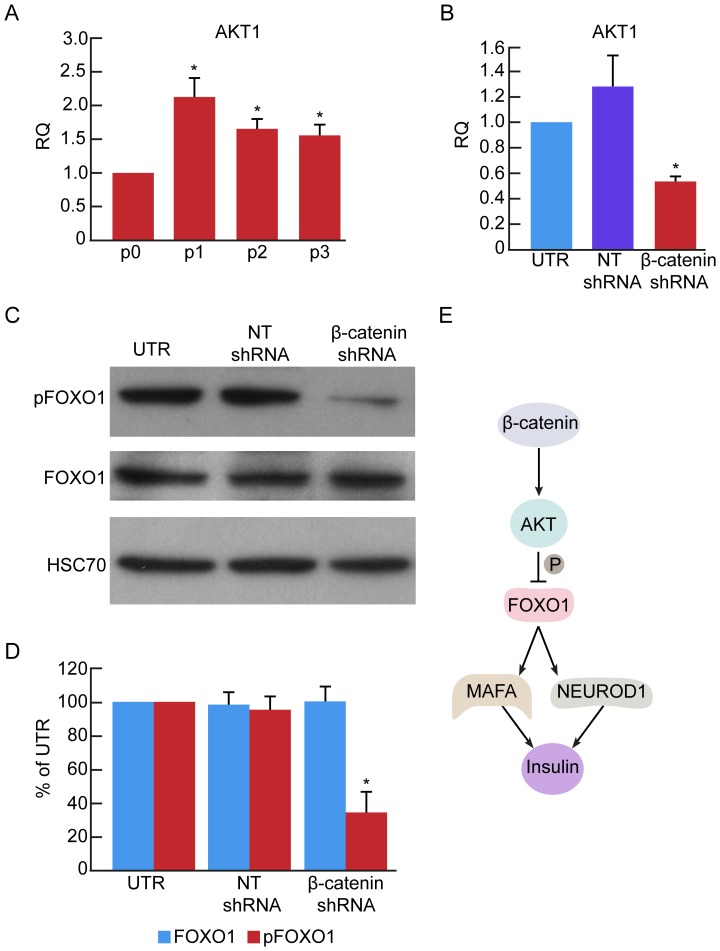
Effect of β-catenin downregulation on expression of AKT1 and FOXO1. **A**, qPCR analysis of RNA extracted from isolated islets (p0) and expanded islet cells at the indicated passage number. Data are mean±SE (n = 4 donors). *p<0.05, relative to p0. **B**, qPCR analysis of RNA extracted from sorted eGFP^+^ BCD cells at passages 6–8, 7 days following infection with β-catenin or NT shRNA viruses. Data are mean±SE (n = 3 donors). *p<0.05, relative to NT shRNA. **C,D**, Immunoblotting of FOXO1 and phospho-FOXO1 in expanded islet cells infected at passages 5–6 with β-catenin or NT shRNA viruses, and analyzed 7 days later. Data are mean±SE (n = 4 donors). *p<0.05, relative to NT shRNA. **E**, Proposed mechanism for activation of insulin transcription by β-catenin downregulation.

To investigate a possible mechanism responsible for activation of insulin expression by β-catenin shRNA treatment, we evaluated the effect of β-catenin downregulation on expression of AKT and FOXO1. β-catenin has been shown to activate *AKT* transcription [25]. AKT in turn phosphorylates FOXO1 and inhibits its activity [26]. FOXO1 is an inducer of the insulin gene transcription factors NEUROD1 and MAFA [27]. RNA analyses revealed a 2-fold increase in AKT1 transcript levels during the first 3 weeks of islet cell expansion in culture (Fig. 6A). Conversely, a 2-fold decrease in AKT1 transcript levels was observed in cells treated with β-catenin shRNA, compared with cells treated with NT shRNA (Fig. 6B). The levels of phosphorylated FOXO1 decreased 3-fold, while no change was detected in total FOXO1 protein levels (Fig. 6C,D). The elevated FOXO1 activity may be responsible for the observed 2-fold increase in *NEUROD1* and *MAFA* transcript levels in BCD cells (Fig. 4D). Taken together, these findings suggest a possible mechanism linking β-catenin downregulation to insulin gene expression (Fig. 6E).

**Figure 7:**
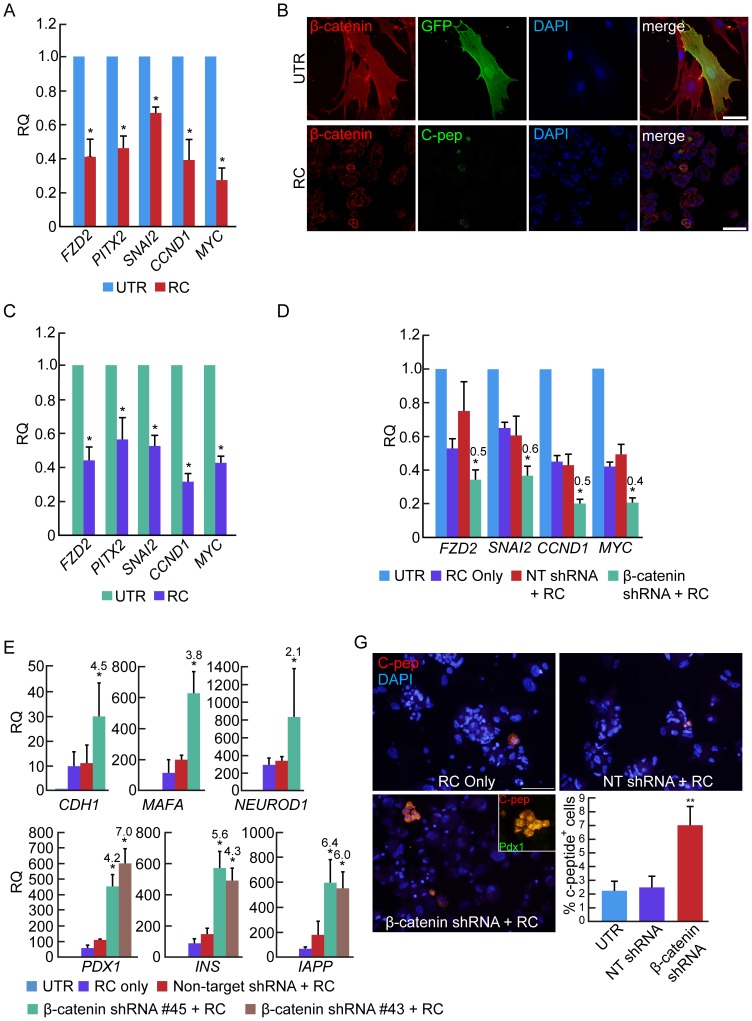
Synergistic effects of β-catenin downregulation and RC treatment. **A**, Effect of a 4-day RC treatment on levels of WNT pathway gene transcripts in expanded islet cells at passages 5–6. Data are mean±SE (n = 4–5 donors). *p<0.05, compared with UTR cells. **B**, Effect of a 4-day RC treatment on subcellular localization of β-catenin in expanded islet cells at passage 6. Beta-catenin is localized throughout the cell in >98% of expanded untreated cells, while >98% of cells treated with β-catenin shRNA show β-catenin membrane localization. Bar = 50 µm. **C**, Effect of a 4-day RC treatment on levels of WNT pathway gene transcripts in sorted eGFP^+^ BCD cells at passages 5–6. Data are mean±SE (n = 3–4 donors). *p<0.05, compared with UTR cells. **D**,**E**, Synergistic effect of a 8-day RC treatment and β-catenin shRNA on levels of WNT pathway target gene (D) and β-cell transcripts (E) in expanded islet cells at passages 5–6. Data are mean±SE (n = 3–5 donors). *p<0.05, relative to nontarget shRNA. **F**, C-peptide immunofluorescence in expanded islet cells infected at passages 5–6 with β-catenin or NT shRNA viruses, and treated for 4 days with RC. Bar = 75 µm. Values are mean±SD (n = 3 donors), based on quantitation of >1000 cells in each group. *p<0.005, relative NT shRNA.

We have previously shown that BCD cells can be redifferentiated by treatment with a combination of soluble factors in serum-free medium, termed Redifferentiation Cocktail (RC) [6]. These factors include activin A, exendin-4, nicotinamide, and high glucose concentrations, which have been shown to promote β-cell differentiation, in serum-free medium supplemented with B27 and ITS. RC treatment resulted in a significant reduction in transcripts encoding WNT pathway receptor and target genes (Fig. 7A), and in translocation of β-catenin from the cytoplasm and nucleus in eGFP^+^-labeled BCD cells to the membrane in redifferentiated C-peptide^+^ cells (Fig. 7B). Analysis of sorted eGFP^+^ BCD cells showed changes in transcripts encoding WNT pathway gene expression similar to those observed in total expanded islet cells (Fig. 7C). Expanded islet cells subjected to both RC treatment and β-catenin shRNA showed a synergistic 2-fold decrease in transcripts encoding FZD2 and WNT pathway target genes, compared with cells treated with RC and NT shRNA (Fig. 7D). A 3.6–7-fold increase in transcripts encoding CDH1, insulin gene transcription factors, insulin, and IAPP, was also observed in cells treated with RC and β-catenin shRNA, compared with those treated with RC and NT shRNA (Fig. 7E). Finally, the number of C-peptide^+^ cells more than doubled following the dual treatment, compared with cells treated with RC alone (Fig. 7F), suggesting that the synergistic effect was manifested in induction of redifferentiation in a larger number of BCD cells, rather than stimulation of higher insulin expression in cells already induced to differentiate. Overall, these findings suggest that a further decrease in WNT pathway activity, compared with that induced by RC alone, results in enhanced BCD cell redifferentiation. A second β-catenin shRNA sequence was used to confirm that the observed changes were due to β-catenin-specific inhibition. RC treatment combined with β-catenin shRNA TRCN-3843, which reduced β-catenin protein levels in HeLa cells by 76%, resulted in an increase in *INS*, *IAPP* and *PDX1* transcripts comparable to that observed using β-catenin shRNA TRCN-3845 (Fig. 7E), indicating that the effect on cell redifferentiation was caused by specific inhibition of β-catenin expression.

**Figure 8:**
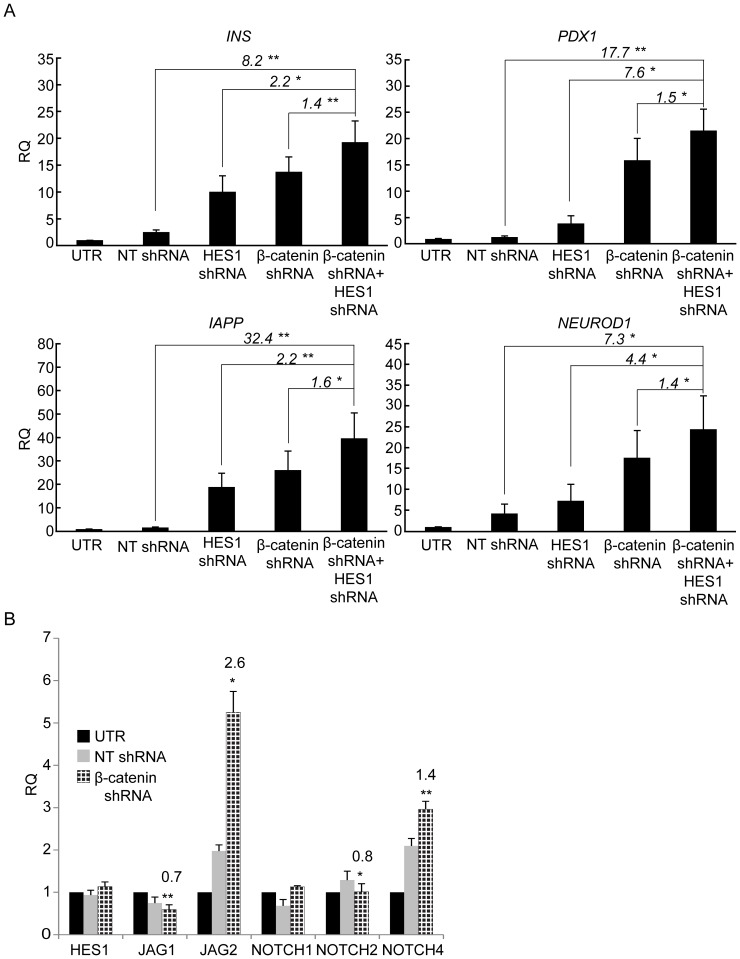
Synegistic effects of β-catenin and HES1 downregulation. **A**, qPCR analysis of RNA extracted from expanded islet cells at passage 6, 7 days following infection with β-catenin shRNA, HES1 shRNA, or both. Data are mean±SE (n = 3 donors). *p<0.05, **p<0.005. **B**, Effect of β-catenin downregulation on levels of NOTCH pathway gene transcripts. qPCR analysis of RNA extracted from expanded islet cells at passages 5–6, 7 days following infection with β-catenin or NT shRNA viruses. Data are mean±SE (n = 3–8 donors). *p<0.05, **p<0.005, relative to NT shRNA.

Our previous work has shown that inhibition of the NOTCH pathway mediator HES1 induces BCD cell redifferentiation [9]. We therefore investigated a possible synergistic effect of inhibiting both β-catenin and HES1 on redifferentiation. As seen in Fig. 8A, a combination of the two treatments resulted in 1.4–7.6-fold higher levels of transcripts encoding insulin, IAPP, and the insulin gene transcription factors PDX1 and NEUROD1. β-catenin has been shown to affect expression of genes encoding NOTCH pathway components [28]. Treatment with β-catenin shRNA resulted in a significant upregulation of JAG2 and NOTCH4 (Fig. 8B), both of which were found to be downregulated in expanded islet cells, compared with human islets [8]. In addition, β-catenin shRNA induced downregulation of NOTCH2, which was upregulated in expanded islet cells, compared with human islets, and of JAG1 (Fig. 8B). Thus, part of the synergistic effect of the two shRNAs on redifferentiation could be due to blocking of β-catenin effects on expression of NOTCH pathway components. Taken together, these findings suggest that simultaneous inhibition of WNT and NOTCH pathways can contribute to BCD cell redifferentiation.

## Discussion

Our findings demonstrate that proliferation and dedifferentiation of BCD cells in culture are associated with activation of the WNT pathway, and that partial inhibition of this pathway is sufficient for induction of BCD cell growth arrest, MET, and redifferentiation. These results are reproducible in cells derived from multiple human donors. WNT pathway activation may be initiated by islet dissociation into single cells, which would release membrane-associated β-catenin into the cytoplasm, from which it can enter the nucleus and activate target gene transcription [29]. This would further donwnregulate CDH1-associated membrane complexes through *CDH1* inhibition. Activation of WNT receptors by WNT ligands present in the serum may further stimulate nuclear β-catenin levels by inhibiting its degradation.

Inhibition of β-catenin expression reduced its cellular levels to <40% of control cells. This reduction was sufficient for a significant decrease in cell proliferation rates, likely mediated by downregulation of *CCND1* and *MYC* expression, which resulted in upregulation of the cell cycle blockers p21 and p27 [30]–[32]. β-catenin inhibition also induced a significant decrease in expression of EMT transcription factors, resulting in elevated CDH1 expression (Fig. 7E) and MET. Since inhibition of β-catenin expression was incomplete, its residual amounts likely sufficed for its function in adherens junction formation, while its transcriptional activities in repressing production of cell cycle blockers, and inducing expression of suppressors of *CDH1* transcription, were significantly inhibited.

Our findings suggest that the mechanism underlying the activation of insulin transcripts in a striking quantitative inverse proportion to the decrease in β-catenin transcripts may involve AKT-FOXO1 interaction. Treatment with β-catenin shRNA resulted in a decrease in AKT transcript levels, which could be responsible for the observed reduction in inactive phospho-FOXO1, likely leading to upregulation of FOXO1 activity. FOXO1 activity has been demonstrated to play important roles in maintenance of mouse β-cell identity [33], [34], induce insulin gene transcription by activating *NEUROD1* and *MAFA* expression, and promote growth arrest by activating expression of *CDKN1B*, encoding the cell cycle blocker p27 [35].

β-catenin shRNA potentiated the redifferentiation effects of RC treatment, which itself downregulates expression of WNT pathway components. This synergistic effect further supports a quantitative inverse correlation between WNT pathway activity and insulin expression in BCD cells. The finding that simultaneous inhibition of both WNT and NOTCH pathways results in a synergistic effect on β-cell gene expression reflect activity of the two pathways through different mediators, and/or potentiation of effects on the same mediators. Alternatively, this synergy may reflect the mutual stimulatory effects these two pathways exert on each other [28].

Taken together, these results suggest that inhibition of the WNT pathway induces significant BCD cell redifferentiation following expansion in vitro, and thus may contribute to therapeutic approaches based on expanding the functional β-cell mass obtained from scarce donor islet tissue for transplantation into multiple recipients. This prospect will require replacement of the β-catenin shRNA viral vector with small-molecule inhibitors of the WNT pathway, functional assessment of the redifferentiated cells in vitro and in vivo, and development of immunoprotective approaches. In addition, inhibition of the WNT pathway may be applicable for reversing β-cell dedifferentiation in vivo, which has been implicated in early stages of both type 1 and type 2 diabetes [33], [36]–[38], thus circumventing the need for β-cell transplantation.

## Supporting Information

Figure S1 **β-catenin shRNA does not induce cell apoptosis.** TUNEL assay of expanded human islet cells at passages 5–6 infected with β-catenin shRNA. Cells treated with DNase I served as positive control. Apoptotic cells were labeled with FITC. Bar = 50 µm.
(TIF)

Figure S2 **β-catenin shRNA does not induce expression of non-β islet cell transcripts in BCD cells.** qPCR analysis of RNA extracted from eGFP^+^ BCD cells at passages 4–5, 7 days following infection with β-catenin or NT shRNA viruses. Data are mean±SE (n = 3 donors). P values are relative to NT shRNA.
(JPG)
