# Supplementary figures and images for: Redifferentiation of Adult Human β Cells Expanded In Vitro by Inhibition of the WNT Pathway
Source: PLoS One. 2014 Nov 13;9(11):e112914. doi: 10.1371/journal.pone.0112914 (PMC4231080; doi:10.1371/journal.pone.0112914)

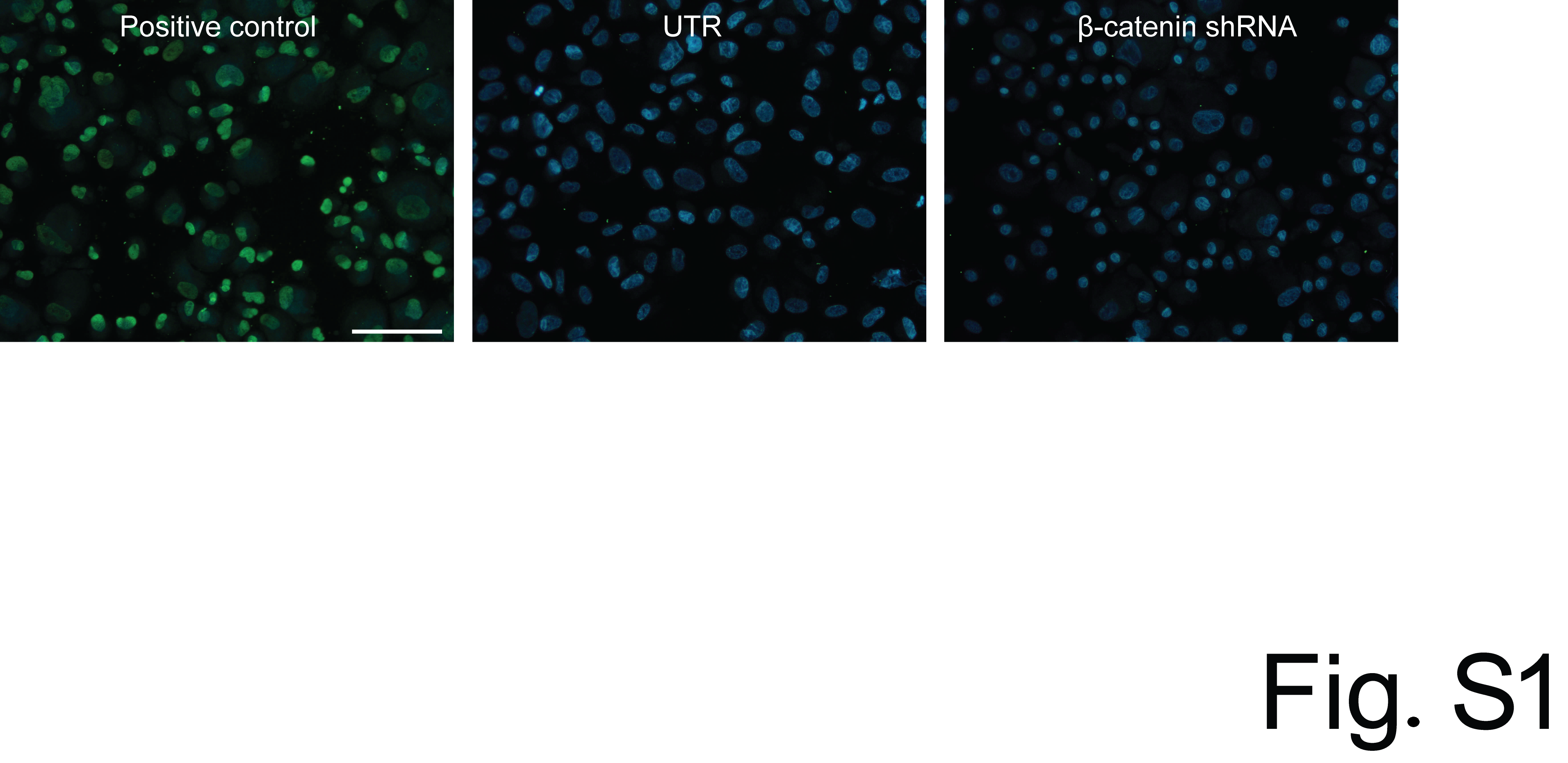

Supplement: Figure S1 — β-catenin shRNA does not induce cell apoptosis. TUNEL assay of expanded human islet cells at passages 5–6 infected with β-catenin shRNA. Cells treated with DNase I served as positive control. Apoptotic cells were labeled with FITC. Bar = 50 µm. (TIF) [file pone.0112914.s001.tif]

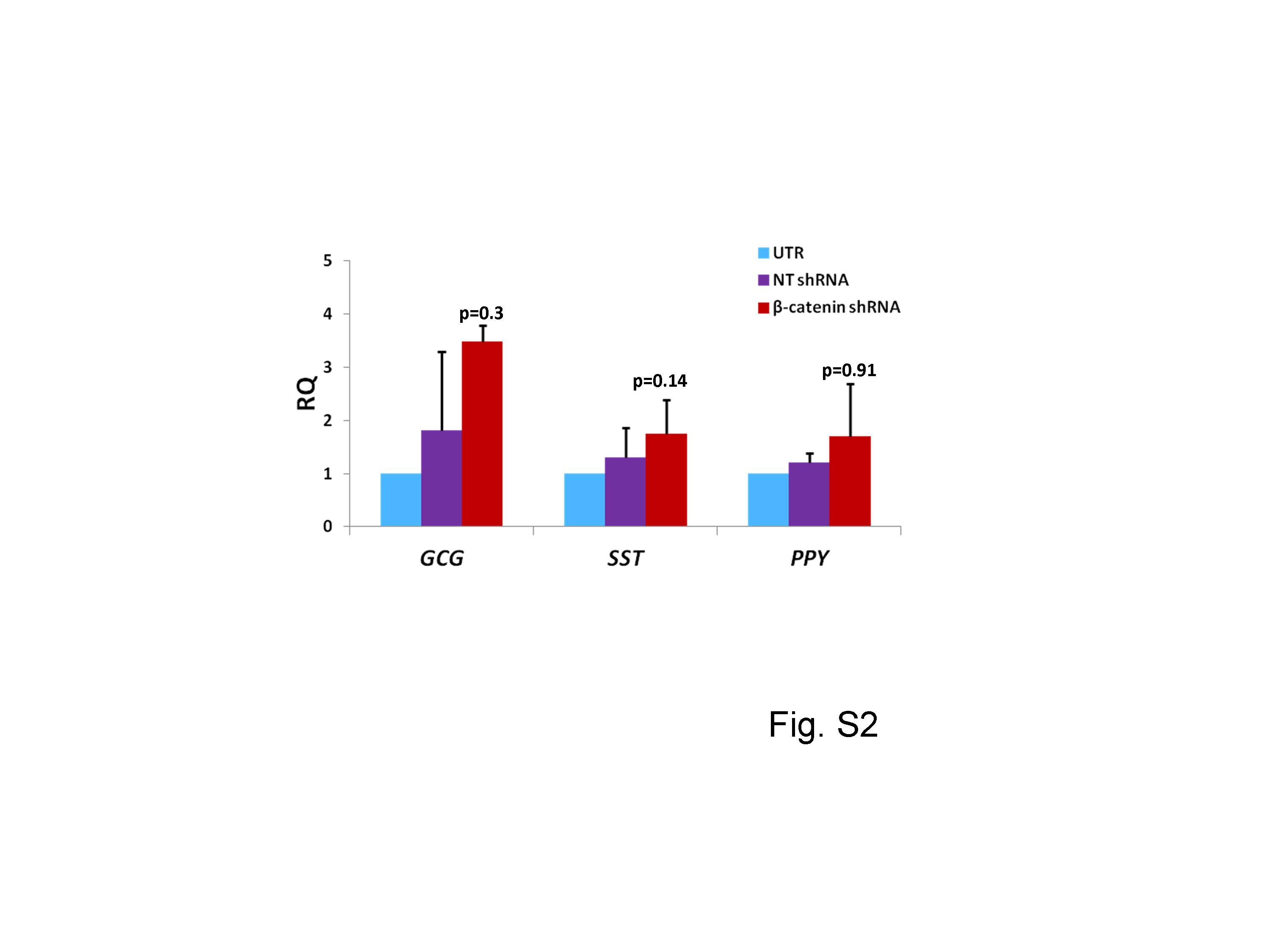

Supplement: Figure S2 — β-catenin shRNA does not induce expression of non-β islet cell transcripts in BCD cells. qPCR analysis of RNA extracted from eGFP+ BCD cells at passages 4–5, 7 days following infection with β-catenin or NT shRNA viruses. Data are mean±SE (n = 3 donors). P values are relative to NT shRNA. (JPG) [file pone.0112914.s002.jpg]
